# Medicinal Plants from North and Central America and the Caribbean Considered Toxic for Humans: The Other Side of the Coin

**DOI:** 10.1155/2017/9439868

**Published:** 2017-11-02

**Authors:** Angel Josabad Alonso-Castro, Fabiola Domínguez, Alan Joel Ruiz-Padilla, Nimsi Campos-Xolalpa, Juan Ramón Zapata-Morales, Candy Carranza-Alvarez, Juan Jose Maldonado-Miranda

**Affiliations:** ^1^Departamento de Farmacia, División de Ciencias Naturales y Exactas, Universidad de Guanajuato, Guanajuato, GTO, Mexico; ^2^Centro de Investigación Biomédica de Oriente, Instituto Mexicano del Seguro Social, Metepec, PUE, Mexico; ^3^Departamento de Sistemas Biológicos, Universidad Autónoma Metropolitana, Unidad Xochimilco, Mexico City, Mexico; ^4^Unidad Académica Multidisciplinaria de la Zona Huasteca, Universidad Autónoma de San Luis Potosí, Ciudad Valles, SLP, Mexico

## Abstract

The consumption of medicinal plants has notably increased over the past two decades. People consider herbal products as safe because of their natural origin, without taking into consideration whether these plants contain a toxic principle. This represents a serious health problem. A bibliographic search was carried out using published scientific material on native plants from Mexico, Central America, and the Caribbean, which describe the ethnobotanical and toxicological information of medicinal plants empirically considered to be toxic. A total of 216 medicinal plants belonging to 77 families have been reported as toxic. Of these plants, 76 had been studied, and 140 plants lacked studies regarding their toxicological effects. The toxicity of 16 plants species has been reported in clinical cases, particularly in children. From these plants, deaths have been reported with the consumption of* Chenopodium ambrosioides*,* Argemone mexicana*, and* Thevetia peruviana*. In most of the cases, the principle of the plant responsible for the toxicity is unknown. There is limited information about the toxicity of medicinal plants used in Mexico, Central America, and the Caribbean. More toxicological studies are necessary to contribute information about the safe use of the medicinal plants cited in this review.

## 1. Introduction

The use of herbal medicine has increased around the world due to its presumptive efficiency, availability, and general acceptance. Approximately 80% of the general population, especially in developing countries, uses medicinal herbs for primary health care [1, 2]. Worldwide, the interest in medicinal plants by patients has increased over the past two decades. The global market for medicinal plants and plant-derived drugs in 2015 was estimated at 25.6 billion dollars and is expected to rise to 35.4 billion dollars in 2020 [3]. This clearly indicates that the consumption of medicinal plants is a current topic of interest. Despite the high consumption of medicinal plants and related products, their toxicity remains to be evaluated. In addition, many medicinal plants require scientific evidence for their medicinal use, particularly those that are sold as food supplements.

Some medicinal plants might trigger undesirable side effects for human health because of (a) pharmacodynamic interaction with prescribed drugs, (b) intrinsic effects, (c) pharmacokinetic interaction with prescribed drugs, and (d) the presence of contaminants and/or pathogenic microorganisms. Other factors that impact the toxicity of medicinal plants in humans include the age of the patient, nutritional status, and the presence of chronic diseases. The concentration of toxic metabolites in plants is determined by the season of collection, nutrients in the soil, and growth stage, among others [4].

The main reasons for focusing this review on medicinal flora with supposed toxicological effects from Mexico, Central America, and the Caribbean are as follows: (a) the ancient importance of traditional medicine in this region, (b) their great biodiversity, and (c) the current use of herbal products. This review will be useful for physicians, toxicologists, pharmacologists, and general audiences. We have tried to describe in detail some toxic symptoms reported with the consumption of the medicinal plants covered in this review.

## 2. Methodology

A bibliographic search was conducted from July 2016 to May 2017 of published scientific material on native plants from Mexico, Central America, and the Caribbean that describes the ethnomedical and toxicological information for medicinal plants reputed to be toxic for humans. The following keywords were searched in different scientific databases: plant extract, toxicity, Mexico, and Central America. Additional data were acquired from undergraduate and postgraduate theses, as well as published and electronic books. The admittance criteria for the selection of scientific information in this review were as follows: (i) plants native to North and Central America and the Caribbean, (ii) plants used for medicinal purposes with or without toxicological studies, (iii) plants with experimental reports on their* in vitro* and/or* in vivo* toxicity, (iv) plants thought to be toxic for humans, (v) studies where the concentrations were presented as weight/volume relationship in international units (mg/ml, *μ*g/ml), (vi) studies where the doses were presented as weight/weight relationship in international units (mg/kg, g/kg), and (vii) plants with information obtained from a clear source. Scientific studies reporting the combination of plant extracts were excluded. Medicinal plants considered toxic were classified into two categories: (1) plants with toxicological evidence reported in a scientific source and (2) plants without toxicological evidence. All plant names and their distributions were confirmed at the Missouri botanical garden [73]. Many of the medicinal plants cited in this review have no common name in English. Therefore, the common names were given in Spanish (Table 1).

## 3. Medicinal Plants Considered to Be Toxic for Humans

A total of 216 medicinal plants belonging to 77 families reported as toxic were recorded. Of these plants, 76 had been studied, and 140 plants lacked studies regarding their toxicological effects (Table 1).* Aristolochia* (6 plant species),* Euphorbia* (6 plant species),* Solanum* (5 plant species), and* Asclepias* (5 plant species) are the plant genera most often reported to induce toxicity (Table 1). Chemotaxonomic studies should be performed to identify the toxic principle in these genera. The parts of the plants considered toxic are listed in the following order: aerial parts including branches, leaves and flowers (22%), whole plant (22%), leaves exclusively (15%), seeds (14%), roots (8%), fruits (8%), bark (4%), latex (3%), and other plant parts.

The signs and symptoms of toxicity induced by medicinal plants are reported in Table 1. The main toxic effects occur in the following order: nausea and vomiting (20%), dermatitis (14%), gastritis (9%), abdominal pain (9%), abortifacient (8%), skin burns (8%), hepatotoxicity (7%), severe diarrhea (6%), cardiotoxicity (5%), nephrotoxicity (2%), numbness (2%), dizziness (2%), and hallucinations (2%), among others.

### 3.1. Dosages

In most of the cases, the dose for the induction of toxic effects by medicinal plants is not indicated. Usually, consumers of medicinal plants believe that increasing the consumption of these products will increase the efficacy of the treatment. In these cases, the daily dosage is exceeded, which triggers toxicity. For instance, the roots of* Ipomoea purga*, a purgative agent, are used at a dose of 2 g/L/day. Administration of higher doses induces vomiting and abdominal pain [15]. Fresh leaves of* Prunus serotina*, used for the treatment of cough, or* Zanthoxylum fagara*, an anxiolytic agent, each must be consumed in a maximum quantity of five leaves in 250 ml of water per day. Higher doses produce spasms and nausea [26]. Approximately 5 mL of an infusion of* Picrasma excelsa* (10 g/L) should be administered three times per day. Higher doses induce hypotension. This infusion should not be prepared with ethanol and orally administered. If a person consumes the hydroalcoholic infusion, the consequences could be lethal [35]. The maximum consumption of* Manilkara zapota* seeds should be 10 seeds per day. A higher consumption of these seeds might induce vomiting and gastroenteritis [26]. On the other hand, Sosa-Gómez [35] recommends the preparation of an infusion using approximately 1–3 g* Argemone mexicana* leaves in 1 L of water. This infusion should be taken 3 times per day. A higher dose might induce immobilization.

Studies analyzing the range of doses considered safe for human consumption remain to be performed. The use of natural products needs scientific evidence to corroborate the medicinal uses attributed to different plant species. Many medicinal plants sold as “food supplements” lack warnings if the suggested dosage is exceeded.

### 3.2. Toxic Principles

In some cases, the toxic principle is known. For instance, it is reported that cefalatin, the main toxic compound in* Cephalanthus occidentalis* bark, induces vomiting, anemia, and seizures, among other toxic effects. Similarly, hederagenin is the main toxic compound in* Clematis dioica*, which is a caustic substance [4]. Monocrotaline is the compound responsible for the toxic effects in* Crotalaria sagittalis*. Cianhidric acid, one of the most toxic compounds in plants, is found in* Crescentia cujete* fruit,* Phaseolus lunatus* whole plant, and* Prunus serotina* leaves and seeds [4]. In* Phaseolus lunatus*, the concentration of cianhidric acid ranges 6.8–533 mg/kg dw [74, 75]. There is limited information on the major toxic compounds cited in this review. Therefore, the identification of toxic principles in medicinal plants is necessary.

## 4. Toxicology

### 4.1. *In Vitro* Studies

The* Artemia salina* (brine shrimp) bioassay has been widely used for the analysis of acute toxicity* in vitro*. Although there are no range values to consider an extract or compound as toxic in the brine shrimp test, vincristine, the positive control for toxicity, has a lethal concentration 50 (LC_50_) = 0.91 *μ*g/ml [76]. Considering this value, plant extracts or compounds with LC_50_ values 1000-fold higher than vincristine could be considered nontoxic.

The following plant extracts have been tested for their* in vitro *toxicology using the brine shrimp test and had LC_50_ values higher than 1000 *μ*g/ml. The ethyl acetate fraction of* Solanum nigrescens* aerial parts [77], the ethanol extract of* Ambrosia peruviana *whole plant [78], the aqueous extract of* Jatropha gossypifolia* aerial parts [79], the methanol extract of* Jatropha dioica* leaves [80], the aqueous extract of* Cnidoscolus urens* whole plant [79], the ethanol extract of* Crescentia cujete* fruits [81], the aqueous extract of* Enterolobium cyclocarpum* bark [82], and the ethanol extract of* Cordia dentata* leaves and their fractions [83].

The plant extract and compounds that could be considered dangerous (LC_50_ = 100–1000 *μ*g/ml) include the following: methanol and hexane proportions derived from a hexane extract of* Gymnosperma glutinosum* aerial parts [84], the ethyl acetate extracts of* Monstera deliciosa* branches [85], and icosandrin, a cyclic homoflavonoid isolated from* Phytolacca icosandra* [86].

The plant extract and compounds that could be considered toxic (LC_50_ = 10–100 *μ*g/ml) include the following: the ethyl acetate extracts of* Monstera deliciosa* leaves [85], the ethanol extract of* Solanum americanum* fruits [87], the ethanol extract of* Scoparia dulcis* aerial parts [76], the methanol extract of* Enterolobium cyclocarpum *leaves [88], the ethanol extract of* Pimenta dioica* leaves [89], and the hydroalcoholic extract of* Sanguinaria canadensis* whole plant [90]. None of the plant extracts or compounds included in this review were considered highly toxic (LC_50_ < 10 *μ*g/ml).

#### 4.1.1. Cytotoxicity

Other plant extracts and their compounds have been tested in other* in vitro* models, including cytotoxicity test in nontumorigenic cells, genotoxicity using the comet assay on lymphocytes, and the mutagenic test using lymphocytes or* Salmonella* spp. The positive controls for cytotoxicity in nontumorigenic cells include cisplatin and Taxol. These compounds have inhibitory concentration 50 (IC_50_) values ranging from 0.1 to 4 *μ*g/ml [91]. Some plants extracts have been reported to lack cytotoxic effects (IC_50_ > 250 *μ*g/ml) in nontumorigenic cells. These include the ethanol extract of* Phoradendron serotinum* leaves tested on peripheral blood mononuclear cells [91], the aqueous extract of* Cnidoscolus chayamansa *leaves on baby hamster kidney (BHK) cells [92], and the aqueous extract of* Enterolobium cyclocarpum* bark assayed on 3T3 murine preadipocytes [82]. Additionally, the ethanol extract of* Equisetum hyemale* aerial parts evaluated on rabbit corneal fibroblasts (SIRC) [93], the methanol extract of* Enterolobium cyclocarpum* leaves evaluated on Vero cells (obtained from kidney epithelial cells extracted from the African green monkey* (Cercopithecus aethiops)* [88], and the diterpene ent‐kaur‐16‐en‐19‐oic acid, obtained from* Annona cherimola*, tested on rat embryo primary striatal cultures [94]. On the other hand, the hydroalcoholic extract of* Hura crepitans* leaves had an IC_50_ = 107.7 *μ*g/ml in lung fibroblasts [95].

#### 4.1.2. Mutagenicity and Genotoxicity

Regarding mutagenicity, parthenin, isolated from* Parthenium hysterophorus*, lacked mutagenicity (0.19 to 19 *μ*M) but showed chromosomal aberrations at concentrations of 10–60 *μ*M in blood lymphocytes [96]. These results suggested genotoxic effects of parthenin. A methanol extract of* Indigofera suffruticosa* aerial parts (1.25–7.5 mg/plate) showed mutagenic activity in a* Salmonella* microsome assay [97]. The acetone extract of* Heliopsis longipes* roots (10–80 *μ*g/Petri dish) and its active compound affinin (6.25–50 *μ*g/Petri dish) were not mutagenic, as evaluated by the Ames test [98]. Lobeline (5–10 mg/kg i.p.), an alkaloid isolated from* Lobelia inflata*, had no genotoxic or mutagenic effects in the comet assay, the micronucleus test in bone marrow, or the Salmonella/microsome mutagenic assay [99].

For genotoxicity, the ethyl acetate/n-hexane extract of* Zinnia peruviana* aerial parts tested using 5 and 20 mg/ml extracts showed genotoxic effects in PBMC compared to the positive control of copper sulfate (1%) [100]. A butanol fraction of* Urera baccifera* roots at a 1.8 mg/g concentration of oxalic acid decreased leukocyte number significantly and increased cell death and DNA damage in primary cultures of leukocytes in comparison to the control treatment [101]. The methanolic extract of* Psittacanthus calyculatus* aerial parts (200 and 400 mg/kg i.p.) did not induce chromosomal damage in peripheral blood erythrocytes obtained from mice after 72 h of exposure [102]. An ethanol extract of* Heliopsis longipes* roots (3–100 mg/kg p.o.) did not produce genotoxic or cytotoxic effects on peripheral blood mononuclear cells obtained from mice 24–96 h after administration [103]. Parthenin, isolated from* Parthenium hysterophorus*, showed genotoxic effects at 4–31 mg/kg i.p. in micronuclei in mouse peripheral blood after 48 and 72 h of treatment [96].

### 4.2. *In Vivo* Acute Studies

#### 4.2.1. Lethal Dose 50 (LD_50_)

The guideline 423 of the Organization for Economic Cooperation and Development (OECD) establishes that substances with an LD_50_ < 5 mg/kg are highly toxic, whereas LD_50_ values from 5 to 50 mg/kg are very toxic, LD_50_ values from 50 to 300 mg/kg are toxic, LD_50_ values from 300 to 2000 mg/kg are dangerous, and LD_50_ values higher than 2000 mg/kg are not dangerous [104].

Some plant extracts showed LD_50_ > 2000 mg/kg p.o. in mice: ethanol extracts of leaves of* Casimiroa edulis* [105] and* Cnidoscolus chayamansa* [106], ethanol extracts of aerial parts of* Moussonia deppeana* [107],* Equisetum hyemale* [93], and* Ruta chalepensis* [108], as well as methanol extracts of leaves of* Chenopodium ambrosioides* [109] and* Rauvolfia tetraphylla* [110]. The same pattern was also shown in aqueous extract of* Cuphea aequipetala* aerial parts [111], ethanol extract of* Plumeria rubra *flowers [112], aqueous extract of* Larrea divaricata* leaves [113], ethanol extract of* Caesalpinia pulcherrima *leaves and bark [114, 115]. aqueous extract of* Euphorbia prostrata* whole plant [116], aqueous-methanol extract of* Ceiba pentandra* leaves [117], petroleum ether, chloroform, and methanol extracts of* Gelsemium sempervirens* roots [118], acetone extract of* Capsicum annum* fruits [119], and aqueous and ethanol extract of* Scoparia dulcis* leaves and whole plant [120, 121].

The following extracts have shown LD_50_ > 2000 mg/kg p.o. in rats: aqueous extract of* Pouteria sapota* seeds [122], methanol extract of* Martynia annua* leaves [123], ethanol extract of* Flourensia cernua* leaves [124], aqueous extract of* Enterolobium cyclocarpum* bark [82], ethanol and aqueous extract* C. pulcherrima* aerial parts [125], aqueous extract of* Passiflora edulis* leaves [126], hydroalcoholic extract of* Magnolia grandiflora* seeds [127], and ethanol extract of* Crescentia cujete* fruits [128]. The same pattern was also shown in methanol extracts of leaves of* Amaranthus spinosus* [129], and* Rauvolfia tetraphylla* [130], chloroform-methanol extract of* Cnidoscolus chayamansa* leaves [131], aqueous extract of* R. humilis* fruits [132], a chloroform fraction from an ethanol extract of* Tagetes erecta* flowers [133], aqueous extract of* Karwinskia humboldtiana* seeds [134], and hydroalcoholic extract of* Senna occidentalis* aerial parts [135], as well as lutein and lutein ester, obtained from* Tagetes erecta* [136], and ethanol extract of* Jatropha gossypiifolia* aerial parts [137], and aqueous extract of* Caladium bicolor* [138].

Other plant extracts showed LD_50_ > 2000 mg/kg i.p. in mice: hexane extract of* Tilia mexicana* inflorescences [139], aqueous extract of* Tagetes lucida* aerial parts [140], ethanol extract of* Mirabilis jalapa *aerial parts [141], and aqueous extract of* Urera baccifera* leaves [142].

Some plant extracts and plant compounds had LD_50_ values from 300 to 2000 mg/kg, which is considered dangerous [104]. These plant extracts were intraperitoneally administered to mice: the ethanol extract of* Tagetes lucida* aerial parts (LD_50_ = 970 mg/kg) [140], the aqueous extract of* Caladium bicolor* leaves (LD_50_ = 1778.28 mg/kg) [138], and the ethanol extract of* Tagetes lucida* aerial parts (LD_50_ = 970 mg/kg i.p.) [140]. On the other hand, the ethanol extract of* Phoradendron serotinum* leaves had an LD_50_ = 375 mg/kg p.o. in mice [143], and sanguinarine, an alkaloid isolated from* Sanguinaria canadensis*, had an LD_50_ = 1658 mg/kg p.o. in rats [144]. Methanol extracts of* Tilia mexicana* inflorescences had LD_50_ values of 375 mg/kg i.p. in mice [139].

Other plant extracts and plant compounds had LD_50_ values varying from 50 to 300 mg/kg, which is considered toxic [104]. Ethanol extract of* Phoradendron serotinum* leaves had an LD_50_ = 125 mg/kg i.p. in mice, [143], whereas capsaicin, the main active principle of* Capsicum annum*, had an LD_50_ = 190 mg/kg p.o. [145]. The acetone extract of* Heliopsis longipes* roots had an LD_50_ = 62.14 mg/kg p.o. in mice, whereas its active compound affinin had an LD_50_ = 113.13 mg/kg p.o. in mice [98]. The ethanol extract of* Heliopsis longipes* roots had an LD_50_ = 288 mg/kg p.o. in mice [103].

The following plant extracts and compounds can be considered very toxic (5–50 mg/kg) [104]: the free alkaloid fraction in hexane and methanol extracts from* Erythrina americana* seeds (LD_50_ = 38.54 to 40.37 mg/kg i.p.) in mice [146],* Jatropha curcas* oil (LD_50_ = 23.34 mg/kg p.o.) in mice [147], and *α*-solamargine, isolated from* Solanum americanum*, with an LD_50_ = 42 ± 2 mg/kg i.p. in rats [148]. The alkaloid N-methylisocorydinium, obtained from* Magnolia grandiflora* trunk wood, had an LD_50_ = 10 mg/kg i.p. in mice [149] and *γ*-coniceine, isolated from* Conium maculatum*, had an LD_50_ = 12 mg/kg p.o in mice [150]. Parthenin, the toxic compound of* Parthenium hysterophorus*, had an LD_50_ = 42 mg/kg i.p. in rats [151]. Capsaicin had an LD_50_ = 8 mg/kg i.p. and 7.80 mg/kg i.m in mice [152]. Sanguinarine, an alkaloid isolated from* Sanguinaria canadensis*, was toxic at 29 mg/kg i.v. in rats [144].

The following plant compounds had LD_50_ values < 5 mg/kg, which is considered highly toxic [104]. Capsaicin had an LD_50_ = 0.56 mg/kg i.v. [152].

#### 4.2.2. Biochemical and Hematological Parameters

Treatment with plant extracts in short-term studies have effects on biochemical and hematological parameters, as well as the levels of the hepatic enzymes alanine aminotransferase (ALT), aspartate aminotransferase (AST), and alkaline phosphatase (ALP). An aqueous extract of* Larrea divaricata* aerial parts (0.5–200 mg/kg i.p.) did not affect the levels of ALT in mice after 2 days [153]. Aqueous extract of* Karwinskia humboldtiana* fruits (1250 mg/kg p.o.) administered to rats for 3 days increased the levels of hepatic enzymes compared to the untreated group [154]. An aqueous extract of* Passiflora edulis* (30 mg/kg p.o.) did not affect motor coordination in mice or change the biochemical measurements in serum after 4 days [155]. *α*-Solamargine (15–35 mg/kg i.p.) did not affect hematological parameters or the levels of hepatic enzymes in rats after 5 days [148], whereas an aqueous extract of* Karwinskia humboldtiana* fruit (5000 mg/kg p.o.) in rats for 5 days induced weight loss (15%) in rats, as well as toxicity in the pancreas [156]. An aqueous extract of* Passiflora edulis* leaves (100–400 mg/kg p.o.) did not affect organ body weight or hematological parameters but decreased the levels of ALT in rats after 7 days of treatment at all doses [126]. A methanol extract of* R. tetraphylla* leaves (1000 mg/kg p.o.) decreased body weight change and food consumption and increased total bilirubin in rats after 7 days [157]. An aqueous extract of* K. humboldtiana* (1000–2000 mg/kg p.o.) administered for 7 days in rats induced alterations in membrane fluidity and ATPase activity in liver submitochondrial particles [158]. An ethanol extract of* Euphorbia hirta* leaves (60.4–483 mg/kg i.p.) [159] and a chloroform fraction from an ethanol extract of* Tagetes erecta* flowers (200–400 mg/kg p.o.) [133] did not affect hematological or biochemical parameters in rats after 14 days. An aqueous extract of* Euphorbia hirta* whole plant administered at a single dose of 2000 mg/kg p.o. significantly decreased the levels of ALP and ALT after 14 days in broiler chickens [160]. Lobeline (5–10 mg/kg i.p.) did not affect the levels of AST, ALT, ALP, and LDH after 4 days of exposure [99].

#### 4.2.3. Toxicity to Reproduction and Pregnancy

*α*-Solamargine (15–35 mg/kg i.p.) did not affect the number of spermatozoa or the weight of the testicles and epididymis of male rats after 24 h of treatment [148]. Jervine (70–300 mg/kg p.o.), a steroidal alkaloid found in* Veratrum californicum*, administered on days 8–10 of gestation induced malformations in the offspring, including isolated cleft palate, mandibular micrognathia, and limb malformations in C57BL/6J and A/J mice [161]. The administration of an aqueous extract of* Ruta chalepensis* leaves (10 mg/kg p.o.) to pregnant rats from day 9 to day 17 of gestation decreased the uterine weight, the number of live fetuses, and the fetal weight. The number of fetal resorptions was also increased, and the fetuses showed skeletal malformations [162]. Additionally, an aqueous extract of* R. chalepensis *leaves (0.8 and 1.6 g/kg p.o.) administered to mice from day 1 to day 14 post coitum caused perinatal changes in mice such as righting reflex, cliff avoidance, and swimming ability, among others [163].

#### 4.2.4. Dermal Tests

An aqueous extract of* Pouteria sapota* seeds lacked dermal irritation in rabbits and showed mild reversible eye irritation in rabbits [122].* Jatropha multifida* sap did not induce skin lesions in rats after 14 days of treatment [164]. A diethyl ether extract of* Jatropha multifida* showed the presence of 16-hydroxyphorbol. This compound showed an irritant dose 50 (ID_50_) of 0.05 *μ*g/ear [165].

### 4.3. *In Vivo* Subacute and Chronic Studies

#### 4.3.1. Biochemical and Hematological Parameters

Some plant extracts and their active compounds have been tested for their effects on biochemical and hematological parameters in rodents for at least 18 days of exposure. A histological study has also been included in some reports. A hydroalcoholic extract* of Senna occidentalis *aerial parts (100–2500 mg/kg p.o.) [135] and a hydroalcoholic extract of* Sapindus saponaria* leaves (44.76 mg/kg p.o.) and fruits (45.0 mg/kg p.o.) [166] did not change the biochemical profile or hematological parameters or alter body weight and organ weight for 30 days in rats. An ethanol extract of the pod of* Plumeria rubra* (50–200 mg/kg p.o) [167] and an aqueous-methanol extract of* Ceiba pentandra* leaves (250 and 500 mg/kg p.o.) [117] did not alter hematological or biochemical parameters in rats and mice, respectively, after 21 days [167]. A chloroform-methanol extract of* Cnidoscolus chayamansa* leaves (1000 mg/kg p.o.) [131] and an ethanol extract of* Moussonia deppeana* aerial parts (1000 mg/kg p.o.) [107] did not affect biochemical or hematological parameters in mice after 28 days of daily administration. In addition, histological examinations of the spleen, kidney, and liver showed no abnormalities. Capsaicin (5–100 mg/kg p.o.) [168], obtained from* Capsicum annum*, and lutein and lutein ester (4–400 mg/kg p.o.) [136], obtained from* Tagetes erecta*, did not affect hematological or biochemical parameters, growth, food consumption, or body weight in mice and rats, respectively, after 28 days. An aqueous extract of* Rivina humilis* fruits (2500 and 5000 mg/kg p.o.) administered daily for 35 days showed no changes in the hematological profile or in the relative organ weight, whereas the same extract administered daily for 90 days at 0.5–2 g/100 g in a powdered diet did not affect hematological parameters, biochemical determinations, or the levels of hepatic enzymes [132].

In contrast, some plant extracts have altered biochemical and/or hematological parameters. An aqueous extract of* Abrus precatorius* leaves (400–1600 mg/kg p.o.) was administered to rats for 18 days. Only the highest dose (1600 mg/kg p.o.) decreased levels of hematological parameters and increased the levels of hepatic enzymes [169]. An aqueous extract of* Scoparia dulcis* leaves (250–500 mg/kg p.o.) showed mild vascular and portal congestion in the heart and the liver, respectively, of rats treated daily with this extract for 30 days. Nevertheless, there were effects in the lungs and testis [120]. A methanol extract of* Rauvolfia tetraphylla* leaves did not affect hematological parameters. However, a significant decrease in the total bilirubin and glucose levels was observed in the mice treated at 100 and 300 mg/kg, with a significant increase in triglycerides at doses of 10–300 mg/kg after 28 days in mice [130]. An ethanol extract of the aerial parts from* Jatropha gossypifolia* (135 mg/kg p.o. or higher doses) reduced the activity in the central nervous system and showed hepatotoxicity, pulmonary damage, and digestive disturbances in rats over 13 weeks of treatment [170]. The lethality was 46.6% and 13.3% among male and female rats under the highest tested dose (405 mg/kg), respectively [170].

#### 4.3.2. Toxicity during Reproduction and Pregnancy

Yao et al. [171] reported that an aqueous extract of* Hydrastis canadensis* (1.86 g/kg p.o.) containing 9.6 mg/ml of berberine and 8.4 mg/ml of hydrastine did not affect fetal development in pregnant rats over 20 days of treatment. A* Prosopis juliflora* seedcase added at 70% to the diet of pregnant rats resulted to be teratogenic (13-fold) compared to the untreated group [172]. Aqueous and ethanol extracts of* Plumeria rubra* pods (200 mg/kg p.o.) had 51% and 100% abortifacient activity, respectively, in female albino rats from day 11 to day 15 of pregnancy [173]. The hydroalcoholic extract of* Lantana camara* leaves (1000–7000 mg/kg p.o.) administered during premating, mating, pregnancy, and lactation (56 days in total) in rats did not affect mating, pregnancy, delivery, and live birth. Nevertheless, the two highest doses tested (3000 and 7000 mg/kg p.o.) produced an increase in the resorption rate and parallel increases in the postimplantation loss index, as well as embryotoxicity characterized by skeletal abnormalities [174].

#### 4.3.3. Carcinogenicity

Only one plant extract has been tested for its carcinogenic effects. Rats (treated with doses ranging 136–1175 mg/kg p.o.) and mice (treated with doses ranging 375–3275 mg/kg) received an aqueous extract of* Hydrastis canadensis* root for 2 years (106 weeks). At the end of the treatment, the doses of 1175 mg/kg in rats and the doses varying from 1120 to 3275 mg/kg in mice showed hepatocarcinoma [175].

### 4.4. Clinical Cases

The toxicity of sixteen species plants has been reported in clinical cases. Fourteen of the sixteen plants are enlisted in Table 2. The other two plants are described in Section 4.4.1. The Naranjo algorithm [176], which consists of 10 questions that address the factors considered to determine the causal relationship in case reports, was used to assess causality. A score is obtained (maximum 13) and the results are classified as doubtful or unlikely (0), possible (from 1 to 4), probable (from 5 to 8), and clear or definite (socre > 9). The event must be definitive from a pharmacological or phenomenological point of view, using, if necessary, a conclusive procedure of reexposure [177].

Those cases that report hepatic damage were also evaluated using the method proposed by the Council for International Organizations of Medical Sciences/Roussel Uclaf Causality Assessment Method (CIOMS/RUCAM) [178], which is an organospecific instrument designed for the assessment of hepatotoxicity. This method evaluates the temporal relationship between the consumption of a substance (drug or natural remedy) and the appearance of hepatic damage, the absence or presence of risk factors, the exclusion of alternative causes of liver injury, among others. The sum of the scores leads to a final score between −8 and 14 points, which results in the following categories: highly probable or definite, probable, possible, or excluded. The amount of information of each clinical case considered for this review was classified as enough (number of criteria: 5-6), regular (number of criteria: 3-4), and poor (number of criteria: 1-2). The following criteria were used to evaluate the amount of information: (1) clear information regarding the intake and time elapsed for the onset of symptoms, (2) information of the dose ingested, (3) explanation and clinical management of the intoxication, (4) information for the exclusion of other causes that might induce the toxic effect, (5) information of the withdrawal of the plant substance, and (6) time of recovery from the toxicity or death of the patient.

The toxicity presented in clinical cases was mainly due to the accidental consumption of toxic medicinal plants, especially by children. In all the cases, the toxic effects occurred after the administration of the plant. The symptoms of toxicity were confirmed using objective evidence. None of the reports provided information about the presence of similar toxic effects compared to a previous experience. Improvement of symptoms occurred in some cases (i.e., [58, 62–64, 69, 71, 72]). The information about the number of ingestion with the plant is only reported in some cases (i.e., [43, 57, 59, 60, 65, 67, 70]).

#### 4.4.1. Case Series

Krenzelok et el. [179] gathered information about* Euphorbia pulcherrima* exposure during an 8-year period in the United States of America. The results showed that children accounted for 93.3% of cases of exposure, which were accidental (98.9% of cases) and by ingestion (94.5% of cases). No deaths were reported. However, this study did not report the symptomatology. The toxicity of* Cimicifuga racemosa* has been extensively studied. The reviews of Borrelli and Ernst [180] and Teschke et al. [181] can be consulted for more information regarding the adverse effects of* Cimicifuga racemosa* in other clinical cases. The prevalence of allergy to* Myroxylon pereirae* resin has been reported in many countries, ranging from 5.4 to 11.8% (i.e., [49, 182–185]). From a total of 27815 patients recorded over 5 years in Croatia, 8.4% were positive to contact dermatitis upon exposure to* Myroxylon pereirae* bark [186]. In another case, the prevalence of toxicity by medicinal plants was also reported.* Jatropha curcas*,* Andira inermis*, and* Canella winterana* were the third, the fourth, and the fifth most cited plant species, respectively, associated with cases of toxicity in Cuba from 1998–2007 [36]. Eddleston et al. [187] reported 351 patients with a history of* T. peruviana* consumption for 2 years. No deaths were reported.

## 5. General Considerations

The identification of the compounds responsible for the toxicity has been reported only in some cases. Urushiol might be the compound responsible for the dermatitis reactions to* Metopium brownei* [188], whereas sanguinarine is the compound associated with the toxicity of* Argemone mexicana*. Eddleston et al. [189] reported six fatalities in patients who ingested between 1 and 10 seeds of* T. peruviana*. These fatalities occurred due to high concentrations of cardiac glycosides (neriifolin, thevetin A, thevetin B, and oleandrin) in seeds. Three toxins (T-544, T-514, and T-496) have been reported in* Karwinskia humboldtiana* [190].* Manihot esculenta*, an important dietary staple, is toxic because of the presence of cyanogenic compounds. Linamarin, the predominant cyanogenic glycoside in* Manihot esculenta*, can be accumulated in a range of concentrations between 100 and 500 mg/kg in roots and leaves. The content of HCN in* Manihot esculenta* has been reported 0.1–1 mg/g fresh weight in the leaves [191]. Several intoxications have been described in humans. The clinical pattern consists of neuropathy and hyperthyroidism [192].

The mechanism of toxicity is also unknown in many cases. The mechanism of toxicity of* Argemone mexicana* oil might be explained by the inhibitory effects of sanguinarine on Na+/K+ATPase, induction of cell membrane damage by reactive oxygen species and lipid peroxidation, and inhibition of DNA polymerase activity, among other effects [193].* Larrea tridentata *and nordihydroguaiaretic acid, its active compound, generate acute hepatotoxicity by the inhibition of cyclooxygenase and cytochrome P-450 [194].

Special attention should be given in medicinal plants such as* Argemone mexicana*,* Chenopodium ambrosioides*, and* Thevetia peruviana*. Effects in humans have been reported due the consumption of these medicinal plants. Other plant species, including* Abrus precatorius, Capsicum annum, Conium maculatum, Erythrina Americana, Heliopsis longipes, Hydrastis canadensis, Jatropha curcas, Jatropha gossypifolia, Karwinskia humboldtiana, Larrea tridentata, Magnolia grandiflora, Parthenium hysterophorus, Phoradendron serotinum, Plumeria rubra, Prosopis juliflora, Ruta chalepensis, Sanguinaria canadensis, Solanum americanum*, and* Veratrum californicum*, have shown effects considered highly toxic, including hepatotoxicity, teratogenic, and cardiotoxicity, or with high toxicity in acute studies. Therefore, a total of 22 plants of 216 cited in this review should be extensively studied in terms of their toxicity. Regarding the hepatotoxicity induced by medicinal plants, Valdivia-Correa [39] reported 15 medicinal plants commonly used in Mexican traditional medicine that induce hepatotoxicity. Five (*Equisetum hyemale*,* Tilia mexicana*,* Passiflora edulis*, and* Scoparia dulcis*) of these fifteen medicinal are considered as toxic, according to our bibliographic research. The induction of hepatotoxicity induced by herbal products represents a serious problem in Mexico since the symptoms and signs might be confused with other diseases, and the diagnosis can be incorrect [39].

Some aspects that influence the toxicity of medicinal plants reported in this study are: (a) time of exposure, (b) misidentification of medicinal plants, and (c) adulteration of medicinal plants. Most of the acute symptoms reported in this review appear in the first 24 h after exposure. Nevertheless, more studies, including subacute and chronic assays, as well as the quantitation of hepatic enzymes, should be performed. In other cases, such as the intoxication of* Crotalaria sagittalis*, the toxic symptoms appear 2 to 6 months after the exposure [4]. However, chronic poisoning induced by medicinal plants is not easily detected since the symptoms are multiple and variable and a diagnosis cannot be made. Many poisonings caused by medicinal plants are classified as of unknown origin because most of the patients deny the consumption of herbal products. For instance, the clinical picture of intoxication with* Karwinskia humboldtiana* might be confused with poliomyelitis [190]. In addition, in most of the cases, the plants are not taxonomically identified [36]. The misidentification of medicinal plants represents a serious problem for human health. The adulteration of medicinal plants sold as food products should be considered as a risk of intoxication by medicinal plants.

Another aspect to consider for further studies is the evaluation of mixtures of medicinal plants and the combination of medicinal plants with allopathic medications. It is thought that the combination of medicinal plants might result in higher beneficial effects compared to those found with single preparations. Nevertheless, it might be the case that two toxic plants are combined and their toxic effects might result in a synergistic action. The self-medication of drugs along with the consumption of medicinal plants is a common practice among patients with chronic diseases [195], which can be considered as an alternative cause of intoxication. In the clinical record, it is not indicated whether the patient consumes medicinal plants. The interaction of herbal extracts and drugs remains to be studied. There are few documented cases that report the toxicity of the combination of plant extracts and drugs. For instance, the combination of* Picrasma excelsa* coumarins are reported to potentiate the activity of warfarin [196]. The toxicity of mixtures of medicinal plants and the combination of medicinal plants with allopathic medication requires further investigation.

## 6. Conclusions

There is limited information about the toxicity of medicinal plants used in Mexico, Central America, and the Caribbean. The molecular mechanisms by which medicinal plants induce toxic effects should also be addressed. In many cases, intoxication by medicinal plants might be confused with other diseases. The detection of intoxication with medicinal plants could be difficult because the symptoms might be confused with other diseases.

The prevention of poisoning in humans can be avoided if the chemical composition of medicinal plants is known.

## Figures and Tables

**Table 1:** Ethnobotanical information of medicinal plants from Mexico and Central America considered as toxic.

Family	Scientific name	Common name in Spanish	Medicinal use	Signs of toxicity [plant part]	Reference
Amaranthaceae	*Amaranthus spinosus* L.^*∗∗*^	Quelite de Puerco	Rheumatism, diuretic, wound healing	Nephrotoxicity [whole plant]	[5]
	*Chenopodium ambrosioides* L.^*∗∗*^	Epazote	Vermifuge, vomit	Nephrotoxicity abortifacient, hepatotoxicity [whole plant]	[6]

Amaryllidaceae	*Allium glandulosum* Link & Otto	Cebolla de monte	Cough, flu, tuberculosis	Numbness, nausea, and vomiting [bulb]	[7]

Anacardiaceae	*Metopium brownei* (Jacq.) Urb.	Chechém negro	Antiviral, Rheumatism	Skin burns [latex from leaves]	[8]
	*Toxicodendron radicans* (L.) Kuntze	Hiedra venenosa	Headache, rheumatism	Dermatitis [latex]	[9]

Annonaceae	*Annona cherimola* Mill.^*∗∗*^	Chirimoya	Diarrhea, dysentery	Abortifacient [aerial parts, fruits]	[10]

Apiaceae	*Conium maculatum* L.^*∗∗*^	Cicuta	Body pain	Hypertension and sweating [whole plant]	[11]

Apocynaceae	*Asclepias curassavica *L.	Rompemuelas	Vermifuge, cancer, wound healing, diuretic	Nausea and vomiting, muscle paralysis [whole plant]	[12]
	*Asclepias linaria *Cav.	Algodoncillo	Cough, fever, purgative, diuretic	Muscle paralysis [leaves]	[13]
	*Asclepias mexicana* Cav.	Venenillo cimarrón	Warts	Numbness [leaves]	[12]
	*Asclepias oenotheroides* Schltdl. & Cham	Hierba lechosa	Tooth ache	Numbness, nausea, and vomiting [leaves]	[5]
	*Asclepias subverticillata* (A. Gray) Vail	Hierba lechosa	Snake bite	Severe diarrhea [leaves]	[5]
	*Plumeria rubra *L.^*∗∗*^	Zacalazúchil	Stomachache, toothache	Dermatitis [latex]	[14]
	*Rauvolfia tetraphylla* L.^*∗∗*^	Cinco negritos	Wound healing, rheumatism	Diarrhea, nausea, and vomiting, hypertension, depression [aerial parts]	[15]
	*Thevetia ahouai *(L.) A. DC.	Bola de venado	Toothache, headache	Cardiotoxicity [fruits and seeds]	[8]
	*Thevetia gaumeri* Hemsl.	Campanilla	Toothache, cancer	Tooth loss [leaves, latex]	[16]
	*Thevetia peruviana* (Pers.) K. Schum.^*∗∗*^	Troncomin	Stomachache	Cardiotoxicity [leaves]	[17]
	*Thevetia thevetioides* (Kunth) K. Schum	Yoyote	Warts, analgesic	Cardiotoxicity [whole plant]	[18]

Aquifoliaceae	*Ilex opaca *Aiton	American holly	Digestive	Cardiotoxicity and vomiting [fruits]	[19]

Araceae	*Anthurium crassinervium *(Jacq.) Schott	Kiilbal chaak	Warts	Dermatitis [sap]	[8]
	*Anthurium pentaphyllum *(Aubl.) G. Don	Hoja de reumatismo de bejuco	Rheumatism	Dermatitis [aerial parts]	[8]
	*Anthurium schlechtendalii *Kunth	Hoja de piedra	Hemorrhage postpartum	Dermatitis [aerial parts]	[8]
	*Caladium bicolor *(Aiton) Vent.^*∗∗*^	Heart of Jesus	Antiseptic	Dermatitis, diarrhea [aerial parts]	[4]
	*Monstera deliciosa *Liebm.^*∗∗*^	Cerimán	Flu, rheumatism	Dysphagia [aerial parts]	[20]

Aristolochiaceae	*Aristolochia foetida *Kunth	Guaco	Snake bite, headache	Hepatotoxicity [whole plant]	[21]
	*Aristolochia grandiflora *Sw.	Flor de pato	Stomachache, snake bite	Abdominal pain, gastritis [roots]	[15]
	*Aristolochia odoratissima* L.	Guaco	Diarrhea, stomachache, belly cramps	Nephrotoxicity and hepatotoxicity [roots]	[22]
	*Aristolochia pentandra* Jacq	Camotillo guaco	Fever, diarrhea	Nephrotoxicity [roots]	[22]
	*Aristolochia reticulata *Nutt.	Texas dutchman's pipe	Digestive	Nephrotoxicity [roots]	[19]
	*Aristolochia serpentaria *L.	Virginia snakeroot	Digestive, diuretic	Nephrotoxicity [roots]	[19]

Asparagaceae	*Yucca filifera* Chabaud	Palma	Cough	Nausea and vomiting [aerial parts]	[14]

Asteraceae	*Acmella repens *(Walter) Rich.	Yerba de San Pedro	Malaria	Hallucinations [aerial parts]	[23]
	*Ambrosia confertiflora* DC.	Amargosa	Diarrhea, vomiting	Gastritis [whole plant]	[13]
	*Ambrosia peruviana* Willd.^*∗∗*^	Altamisa	Rheumatism, pain, fever	Neurotoxicity [whole plant]	[24]
	*Ambrosia psilostachya* DC.	Estafiate	Stomachache	Nausea and vomiting [whole plant]	[25]
	*Artemisia ludoviciana *subsp. mexicana (Willd. ex Spreng.) D.D. Keck	Estafiate	Vermifuge, fever	Numbness, carcinogenic [aerial parts]	[14]
	*Barkleyanthus salicifolius *(Kunth) H. Rob. & Brettell	Jaral Amarillo	Fever, diuretic, rheumatism	Hepatotoxicity [whole plant]	[14]
	*Conyza filaginoides* (DC.) Hieron.	Simonillo	Stomachache, diabetes, anxiolytic	Nausea and vomiting [whole plant]	[7]
	*Eupatorium odoratum* L.	Rama de la cruz	Wound healing, anti-inflammatory	Nausea and vomiting [whole plant]	[5]
	*Flourensia cernua* DC.^*∗∗*^	Hojasén	Stomachache, diarrhea	Hepatotoxicity [leaves]	[13]
	*Gymnosperma glutinosum* (Spreng.) Less.^*∗∗*^	Tatalencho	Diuretic, rheumatism, analgesic	Sleepiness, muscle paralysis [seeds, leaves]	[5]
	*Haplopappus gymnocephalus *DC.	Arnica morada	Body pain, hemorrhoids	Gastritis [aerial parts]	[14]
	*Helenium mexicanum *Kunth	Cabezona	Flu	Gastritis and vomiting [flowers]	[15]
	*Heliopsis longipes *(A. Gray) S.F. Blake^*∗∗*^	Chilcuague	Analgesic	Narcotic [roots]	[26]
	*Montanoa tomentosa *Cerv.	Zoapatle	Rheumatism, cough, menstrual colic	Abortifacient, respiratory failure [aerial parts]	[27]
	*Packera aurea* (L.) Á. Löve & D. Löve	Life root	Amenorrhea, menopause and leucorrhea.	Hepatotoxicity [aerial parts]	[19]
	*Packera candidissima *(Greene) W.A. Weber & Á. Löve	Chuca	Cough	Hepatotoxicity [aerial parts]	[28]
	*Parthenium hysterophorus *L.^*∗∗*^	Escoba amarga	Stomachache, headache	Hypotensive, bradycardia [whole plant]	[29]
	*Parthenium incanum* Kunth	Mariola	Stomachache, diarrhea	Nausea and vomiting [whole plant]	[13]
	*Psacalium decompositum* (A. Gray) H. Rob. & Brettell	Matarique	Diabetes, rheumatism	Neurotoxicity [roots]	[30]
	*Tagetes erecta *L.^*∗∗*^	Flor de muerto (cempaxochitl)	Diarrhea, vermifuge, diabetes, rheumatism	Gastritis [flowers]	[31]
	*Tagetes lucida* Cav^*∗∗*^	Pericón	Stomachache, diarrhea, vomit	Abortifacient [whole plant]	[32]
	*Zinnia peruviana *(L.) L.^*∗∗*^	Mal de ojo	Stomachache, diarrhea	Eye irritant	[14]

Berberidaceae	*Berberis moranensis *Schult. & Schult. f.	Palo amarillo	Rheumatism	Numbness, nausea, and vomiting [aerial parts]	[33]
	*Caulophyllum thalictroides *(L.) Michx.^*∗∗*^	Blue cohosh	Dysmenorrhea, rheumatism	Nausea and vomiting, gastritis [seeds and roots]	[11]
	*Podophyllum peltatum *L.^*∗∗*^	Mayapple	Genital warts	Altered mental states, tachypnea, peripheral neuropathy, nausea and vomiting, hypotension, and fever [whole plant]	[19]

Bignoniaceae	*Crescentia alata *Kunth	Cuatecomate	Cough, asthma	Vomiting, abdominal pain [fruit]	[21]
	*Crescentia cujete* L.^*∗∗*^	Güiro	Cough, tuberculosis	Abortifacient, severe diarrhea [fruits]	[18]

Boraginaceae	*Cordia dentata *Poir.^*∗∗*^	Uvita	Cough	Severe diarrhea [fruits]	[8]
	*Heliotropium curassavicum *L.	Alacrancillo	Asthma, anemia, snake bite	Hepatitis [whole plant]	[11]

Bromeliaceae	*Bromelia pinguin *L.	Piñuela	Cough	Dermatitis [fruits]	[34]
	*Bromelia plumieri *(E. Morren) L.B. Sm.	Timbiriche	Inflammation	Dermatitis [fruits]	[8]

Cactaceae	*Cereus marginatus* DC.	Oregano de zopilote	Rabies	Cardiotoxicity [aerial parts]	[13]
	*Coryphantha pycnacantha *(Mart.) Lem.	Falso peyote	Rheumatism, analgesic	Sedation in tongue [fruit]	[14]
	*Lophophora williamsii* (Lem. ex Salm-Dyck) J.M. Coult.	Peyote	Rheumatism, analgesic	Hallucinations [whole plant]	[30]

Campanulaceae	*Lobelia cardinalis L.*	Lobelia	Cough, flu	Hypothermia, vomiting, abdominal pain [aerial parts]	[4]
	*Lobelia inflata *L.^*∗∗*^	Lobelia	Asthma, muscle relaxant	Hypotension [whole plant]	[35]

Canellaceae	*Canella winterana* (L.) Gaertn.	Cúrbana	Rheumatism, stomachache	Edema, hemorrhage [whole plant]	[36]

Caprifoliaceae	*Lonicera periclymenum *L.	Woodbine	Diuretic, cough	Cardiotoxicity and neurotoxicity [fruits]	[11]
	*Symphoricarpos albus *(L.) S.F. Blake	Snowberry	Tuberculosis	Nausea and vomiting, abdominal pain [whole plant]	[11]

Celastraceae	*Celastrus scandens *L.	Falsa dulcamara	Diuretic, tuberculosis	Gastritis, nausea and vomiting, diarrhea [fruit]	[11]
	*Euonymus atropurpureus *Jacq.	Wahoo	Purgative	Vomiting [bark]	[11]

Commelinaceae	*Commelina elegans* Kunth	Hierba del pollo	Conjunctivitis	Edema, dermatitis [whole plant]	[8]
	*Tradescantia spathacea *Sw.	Maguey morado	Cancer, wound healing, asthma, cough	Skin burns [sap]	[8]

Convulvulaceae	*Ipomoea murucoides* Roem. & Schult.	Cazahuate	Hair loss, wound healing, cough, diuretic	Gastritis [bark]	[37]
	*Ipomoea purga* (Wender.) Hayne	Raíz de Jalapa	Purgative	Vomiting and abdominal pain [roots]	[15]
	*Ipomoea stans* Cav.	Tumbavaqueros	Epileptic seizures	Neurotoxicity [roots]	[38]
	*Ipomoea tricolor *Cav.	Hiedra de flores grandes	Analgesic	Hallucinations [aerial parts]	[14]
	*Turbina corymbosa *(L.) Raf.	Flor de pascua	Fever, wound healing	Hallucinations [seeds]	[4]

Coriariaceae	*Coriaria ruscifolia* L.	Huique	Pneumonia	Hallucinations [aerial parts]	[33]

Cucurbitaceae	*Apodanthera undulata *A. Gray	Gualaista	Gastritis	Vomiting and abdominal pain [roots and seeds]	[14]

Dioscoreaceae	*Dioscorea floribunda *M. Martens & Galeotti	Barbasco amarillo	Rheumatism, body pain	Abortifacient [roots]	[8]

Equisetaceae	*Equisetum hyemale *L.^*∗∗*^	Carricillo	Abdominal pain, urinary tract infections	Hepatotoxicity [whole plant]	[39]

Ericaceae	*Arbutus arizonica *(A. Gray) Sarg.	Madroño	Diuretic	Nausea and vomiting [fruit]	[14]
	*Comarostaphylis discolor *(Hook.) Diggs	Madroño	Diuretic	Nausea and vomiting [fruit]	[33]
	*Kalmia latifolia *L.	Ivy brush	Syphilis	Neurotoxicity, cardiotoxicity [aerial parts]	[11]

Euphorbiaceae	*Acalypha monostachya *Cav. Cav	Hierba del cancer	Cancer	Skin burns [latex]	[14]
	*Adelia barbinervis* Schltdl. & Cham.	Espino blanco	Body pain, wounds	Dermatitis [aerial parts]	[8]
	*Cnidoscolus chayamansa* McVaugh^*∗∗*^	Chaya	Diabetes	Dermatitis [aerial parts]	[8]
	*Cnidoscolus souzae* McVaugh	Ch'iinchay	Rheumatism	Dermatitis [aerial parts]	[8]
	*Cnidoscolus urens* (L.) Arthur^*∗∗*^	Ortiga	Diuretic	Hypotension, skin burns, nausea and vomiting [whole plant]	[24]
	*Croton ciliatoglandulosus* Ortega	Hierba de la cruz	Constipation	Gastritis, excessive salivation [whole plant]	[4]
	*Croton humilis *L.	ik'ja'aban	Wounds	Skin burns [aerial parts]	[8]
	*Dalechampia scandens *L.	Mo'ol koj	Headache	Edema, dermatitis [aerial parts]	[11]
	*Euphorbia cotinifolia *L.	Lechero rojo	Purgative	Skin burns [seeds]	[24]
	*Euphorbia hirta L.* ^*∗∗*^	Hierba de la golondrina	Stomachache	Skin burns [aerial parts]	[14]
	*Euphorbia maculata *L.	Hierba de la golondrina	Tooth ache	Severe diarrhea, vomiting [seeds]	[4]
	*Euphorbia prostrata *Aiton^*∗∗*^	Hierba de la golondrina	Pain in the kidney, wounds, diarrhea	Gastritis, abdominal pain [whole plant]	[31]
	*Euphorbia pulcherrima* Willd. ex Klotzsch^*∗∗*^	Noche buena	Wound healing	Vomiting, diarrhea, abdominal pain [whole plant]	[40]
	*Euphorbia tithymaloides *L.	Redbird flower	Asthma, skin cancer, warts	Irritation of the mouth and throat, vomiting [whole plant]	[20]
	*Hura crepitans* L.^*∗∗*^	Catahua	Laxative	Skin burns [seeds]	[41]
	*Hura polyandra *Baill.	Haba	Stomachache, body pain,	Skin burns [latex], nausea and vomiting, gastritis [seeds and fruits]	[15]
	*Jatropha curcas* L.^*∗∗*^	Piñon	Fever, warts	Dermatitis, vomiting, diarrhea [seeds, leaves]	[36]
	*Jatropha dioica* Sessé^*∗∗*^	Sangre de grado	Cancer, rheumatism, hair loss, wound healing	Dermatitis, vomiting, muscle paralysis [stem, fruits]	[13]
	*Jatropha gossypiifolia* L.^*∗∗*^	Tua tua	Cough, flu, fever	Skin burns, abortifacient [seeds, leaves]	[42]
	*Jatropha multifida *L.^*∗∗*^	Palmeado	Wound healing, to purify blood	Severe diarrhea [seeds]	[43]
	*Manihot esculenta* Crantz^*∗∗*^	Yuca	Wound healing, vermifuge	Poisoning and neurotoxicity [leaves]	[41]
	*Tragia nepetifolia *Cav.	Ortiguilla	Diuretic	Skin burns [Aerial parts]	[44]
	*Tragia yucatanensis* Millsp.	P'oop'ox	Rheumatism	Dermatitis [aerial parts]	[8]

Fabaceae	*Abrus precatorius *L^*∗∗*^	Semilla de culebra	Diabetes, asthma	Stomachache, diarrhea [aerial parts]	[4]
	*Andira inermis *(W. Wright) Kunth ex DC.	Yaba	Vermifuge	Vomiting, fever, hypotension, mental confusion, respiratory insufficiency [bark]	[36]
	*Astragalus plattensis *Nutt.	Garbancillo	Diuretic	Vomiting [leaves]	[26]
	*Caesalpinia pulcherrima* (L.) Sw.^*∗∗*^	Clavellina colorada	Fever, pain, cough	Dermatitis and neurotoxicity [Aerial parts]	[18]
	*Calliandra grandiflora* (L'Hér.) Benth.	Cabello de ángel	Fever	Dermatitis [leaves]	[45]
	*Calliandra molinae *Standl.	Palo de corcho	Hypertension	Hepatotoxicity, hypotension [leaves]	[46]
	*Crotalaria pumila *Ortega	Tronador	Cough, diabetes	Abdominal pain, nausea and vomiting [whole plant]	[14]
	*Crotalaria sagittalis *L.	Cocuite	Fever, snake bite	Anoxia, gastritis, abdominal pain, blood in feces [Aerial parts]	[4]
	*Dalea bicolor *Humb. & Bonpl. ex Willd.	Engordacabra	Diarrhea	Vomiting [aerial parts]	[14]
	*Diphysa robinioides *Benth.	Flor de gallito	Fever, headache	Nausea and vomiting [leaves]	[47]
	*Entada polystachya *(L.) DC.	Bejuco de agua	To promote hair growth	Abdominal pain and severe diarrhea [Fruits]	[4]
	*Enterolobium cyclocarpum *(Jacq.) Griseb.^*∗∗*^	Cascabel sonaja	Bronchitis, sore throat	Severe diarrhea and abdominal pain [Aerial parts]	[4]
	*Erythrina americana* Mill^*∗∗*^	Colorin	Diuretic	Immobilization, hypotension, and respiratory paralysis [seeds]	[15]
	*Erythrina standleyana *Krukoff	Cancer	Cancer	Somnolence, vomit [aerial parts]	[8]
	*Gliricidia sepium* (Jacq.) Kunth ex Walp	Matarratón	Vermifuge	Nausea and vomiting [roots, leaves, seeds]	[24]
	*Indigofera microcarpa *Desv.	Yaga-cohui-pichacha	Purgative	Severe diarrhea [leaves]	[4]
	*Indigofera suffruticosa *Mill.^*∗∗*^	Añil	Vermifuge	Severe diarrhea [leaves]	[4]
	*Leucaena esculenta* (Moc. & Sessé ex DC.) Benth.	Guaje	Wound healing	Nausea and vomiting [seeds]	[48]
	*Myroxylon pereirae *(Royle) Klotzsch^*∗∗*^	Indian balsam	Burns, wounds and ulcers	Allergies, contact urticaria and dermatitis [resin]	[49]
	*Phaseolus lunatus *L.	Frijol ancho	Fever, headache	Seizures, immobilization [whole plant]	[4]
	*Prosopis juliflora *(Sw.) DC.	Mezquite	Fever, diabetes	Nausea and vomiting [seeds]	[4]
	*Robinia pseudoacacia *L.	Black locust	Diuretic, laxative	Anorexia, hypothermia, dyspnoea [bark, leaves and seeds]	[4]
	*Senna multiglandulosa* (Jacq.) H.S. Irwin & Barneby	Parral	Diabetes	Abortifacient [fruit]	[33]
	*Senna occidentalis *(L.) Link^*∗∗*^	Frijolillo	Fever	Gastritis, dermatitis, and conjunctivitis [fruit]	[4]

Garryaceae	*Garrya ovata *Benth.	Cuauhchichic	Diarrhea	Muscle paralysis [bark]	[26]

Gelsemiaceae	*Gelsemium sempervirens* (L.) J. St.-Hil.^*∗∗*^	Retama	Stomachache, asthma, headache, rheumatism	Sedative, vertigo, hypotension, blurred vision [whole plant]	[10]

Gesneriaceae	*Moussonia deppeana *(Schltdl. & Cham.) Hanst.^*∗∗*^	Cacahuapaxtle	Diuretic, gastritis	Abortifacient [aerial parts]	[50]

Lamiaceae	*Hedeoma drummondii *Benth.	Drummond's false pennyroyal	Muscle relaxing	Abortifacient [whole plant]	[26]
	*Hedeoma pulegioides *(L.) Pers.	American pennyroyal	Antispasmodic, pneumonia	Abortifacient, kidney toxicity [aerial parts]	[19]
	*Salvia leucantha* Cav	Lana	Cough, stomachache	Abortifacient [aerial parts]	[27]
	*Satureja brownei* (Sw.) Briq.	Poleo	Colic, cough	Nausea and vomiting, dermatitis, bleeding [whole plant]	[24]
	*Scutellaria lateriflora *L.	Scullcap	Nervousness, headache, fever, anxiety	Giddiness, stupor, confusion, twitching of the limbs, intermission of the pulse [whole plant]	[19]

Loasaceae	*Mentzelia hispida *Willd.	Pegajilla	Rheumatism, anemia	Vomiting [resin]	[14]

Loranthaceae	*Psittacanthus calyculatus *(DC.) G. Don^*∗∗*^	Muerdago o injerto	Hypertension, seizures, rheumatism, wound healing	Nausea and vomiting [whole plant]	[26]

Lythraceae	*Cuphea aequipetala* Cav.^*∗∗*^	Hierba del cáncer	Cancer, wound healing	Numbness, nausea and vomiting [aerial parts]	[10]

Magnoliaceae	*Magnolia grandiflora *L.^*∗∗*^	Magnolia	Nervousness, menstrual colics	Dermatitis [leaves]	[51]

Malpighiaceae	*Malpighia glabra* L.	Acerola	Dysentery, fever	Dermatitis [fruits]	[19]

Malvaceae	*Ceiba pentandra* (L.) Gaertn.^*∗∗*^	Ceiba	Diuretic, cough, fever	Dermatitis [seeds]	[24]
	*Tilia mexicana *Schltdl.^*∗∗*^	Tilia	Nervousness, menstrual pain	Hepatotoxicity [flower]	[39]

Marantaceae	*Thalia geniculata *L.	Kento	Anaemia, hemorrhoids	Edema, gastritis [aerial parts]	[8]

Martyniaceae	*Martynia annua* L.^*∗∗*^	Uña de gato	Snake bite	Nausea and vomiting [seeds]	[18]

Melanthiaceae	*Veratrum californicum *Durand^*∗∗*^	California corn lily	Cancer	Neurotoxicity [whole plant]	[11]
	*Trillium erectum *L.	Bethroot	To prevent obstetric hemorrhage	Skin burns [leaves]	[19]

Menispermaceae	*Menispermum canadense *L.	Canada moonseed	Warts	Cardiotoxicity [fruits]	[11]

Myrtaceae	*Pimenta dioica *(L.) Merr.^*∗∗*^	Pimiento	Rheumatism, stomachache	Neurotoxicity [whole plant]	[19]

Nartheciaceae	*Aletris farinosa *L.	Unicorn root	Laxative, diarrhea, rheumatism	Narcotic [whole plant]	[19]

Nyctaginacea	*Mirabilis jalapa *L^*∗∗*^	Maravilla	Rheumatism, stomachache, fever	Severe diarrhea [roots and seeds]	[4]

Oxalidaceae	*Oxalis alpina* (Rose) Rose ex R. Knuth	Acedera	Gastritis	Poisoning [whole plant]	[7]

Papaveraceae	*Argemone mexicana* L.^*∗∗*^	Cardo santo	Wound healing, fever, diuretic, analgesic	Dermatitis, abortifacient immobilizing, neurotoxicity [whole plant]	[35]
	*Sanguinaria canadensis* L.^*∗∗*^	Bloodroot	Skin cancer, polyps and warts	Narcotic [root]	[19]

Passifloraceae	*Passiflora caerulea* L.	Pasionaria azul	Epilepsy, anxiolytic	Nausea and vomiting, dizziness [flower]	[24]
	*Passiflora edulis* Sims^*∗∗*^	Maracuya	Relaxing	Hallucinations, dizziness, confusion, ataxia, nausea and vomiting, drowsiness, and tachycardia; [flower]	[39, 41]
	*Passiflora quadrangularis* L.	Badea	Vermifuge, obesity	Nausea and vomiting, dizziness [leaves and seeds]	[24]

Petiveriaceae	*Rivina humilis *L.^*∗∗*^	Coralillo	Varicose veins, snake bite, wound healing, stomachache	Nausea and vomiting, abdominal pain [whole plant]	[11]

Phytolaccaceae	*Phytolacca americana* L.	Hierba carmine	Warts	Blurred vision, vomit, vertigo [whole plant]	[52]
	*Phytolacca icosandra *L.^*∗∗*^	Mazorquilla	Cancer, vermifuge, rheumatism	Blurred vision, vomiting, [roots]	[14]
	*Phytolacca rivinoides *Kunth & C.D. Bouché	Mazorquilla	Headache, wound healing, vermifuge	Blurred vision, vomit, vertigo [whole plant]	[30]

Plantaginaceae	*Scoparia dulcis *L.^*∗∗*^	Culantrillo	Diarrhea, stomachache, asthma, nervousness	Hepatotoxicity [whole plant]	[39]

Plumbaginaceae	*Plumbago pulchella *Boiss.	Jiricua	Diabetes, wound healing	Skin burns, vomiting [whole plant]	[14]

Polygalaceae	*Monnina schlechtendaliana* D. Dietr.	Aguacatillo	Dehydration	Nausea and vomiting [fruit]	[33]
	*Polygala senega *L.	rattlesnake root	Cough, diuretic	Gastritis [roots]	[19]
	*Rumex hymenosepalus *Torr.^*∗∗*^	Canaigre	Wound healing	Vomiting, abdominal pain [Aerial parts]	[19]

Ranunculaceae	*Actaea alba *(L.) Mill.	White cohosh	Arthritis, rheumatism, dysmenorrhea	Headache, vomiting, delirium, circulatory failure [whole plant]	[19]
	*Actaea rubra *(Aiton) Willd.	Baneberry	Dysmenorrhea	Vomiting, abdominal pain, salivation [fruits and roots]	[11]
	*Anemone canadensis *L.	Meadow anemone	Body pain and wound healing	Salivation, abdominal pain, and salivation [whole plant]	[11]
	*Cimicifuga racemosa* (L.) Nutt.^*∗∗*^	Baneberry	To manage some symptoms of menopause	Vomiting, abdominal pain [fruit]	[19]
	*Clematis dioica* L.	Barba de chivo	Rheumatism, cough, diuretic	Skin burns [leaves]	[10]
	*Clematis virginiana *L.	Old man's beard	For skin disorders (sores, cuts), itching and venereal eruptions	Dizziness, confusion, profuse salivation [leaves]	[19]
	*Hydrastis canadensis *L.^*∗∗*^	Sello dorado	Laxative	Gastritis, abortifacient [rhizome]	[35]
	*Ranunculus geoides* Humb. Bonpl. & Kunth ex DC	Pata de león	Cough	Skin burns [seeds and fruits]	[30]
	*Thalictrum strigillosum* Hemsl.	Hierba del zorro	Cough	Nausea and vomiting [whole plant]	[7]

Rhamnaceae	*Karwinskia humboldtiana *(Schult.) Zucc.^*∗∗*^	Tullidora	Wound healing	Immobilization, abortifacient [fruit]	[53]
	*Karwinskia mollis *Schltdl.	Capulín	Wound healing	Immobilization [fruit]	[54]
	*Rhamnus kcalifornica *Eschsch	California buckthorn	Laxative	Gastritis [fruit and bark]	[11]

Rosaceae	*Prunus serotina* Ehrh.	Capulín blanco	Cough, diarrhea, abdominal pain	Spasm, nausea and vomiting [leaves and seeds]	[7]

Rubarwiaceae	*Carapichea ipecacuanha *(Brot.) L. Andersson	Ipecac	Dysentery	Rhinitis or asthma [whole plant]	[19]
	*Cephalanthus occidentalis *L.	Guayabillo	Fever	Vomiting, seizures, anemia [bark]	[4]
	*Cinchona pubescens *Vahl^*∗∗*^	Quino	Malaria, varicose veins, internal hemorrhoids	Hypoglycemia, hematologic disorders, urticaria, contact dermatitis, and other hypersensitivity reactions.	[19]

Rutaceae	*Casimiroa edulis *La Llave & Lex.^*∗∗*^	Matasanos	Hypertension, diabetes, rheumatism	Hepatotoxicity, peptic ulcer, hypotensive [leaves]	[46]
	*Ruta chalepensis* L.^*∗∗*^	Ruda	Analgesic	Dermatitis, abortifacient [aerial parts]	[18]
	*Zanthoxylum fagara *(L.) Sarg.	Colima	Nervousness	Narcotic [leaves]	[26]

Santalaceae	*Phoradendron bolleanum* (Seem.) Eichler	Muerdago	Diuretic	Nausea and vomiting [leaves and seeds]	[55]
	*Phoradendron quadrangulare *(Kunth) Griseb.	Muerdago	Cancer	Nausea and vomiting, dehydration [aerial parts]	[11]
	*Phoradendron serotinum *(Raf.) M.C. Johnst.^*∗∗*^	Muerdago	Cancer, diabetes	Nausea and vomiting, abdominal pain, dehydration [aerial parts]	[11]

Sapindaceae	*Sapindus saponaria *L.^*∗∗*^	Jaboncillo	Diuretic	Skin burns [seeds]	[24]

Sapotaceae	*Pouteria sapota *(Jacq.) H.E. Moore & Stearn^*∗∗*^	Zapote	To eliminate louse	Nausea and vomiting, dizziness [seeds]	[35]

Scrophulariaceae	*Buddleja marrubiifolia *Benth.	Azafran de campo	Diuretic	Neurotoxicity [seeds]	[26]

Simaroubaceae	*Picrasma excelsa *(Sw.) Planch.	Palo Amarillo	Stomachache, diabetes	Hypotension [bark]	[35]
	*Castela tortuosa* Liebm.	Chaparro amargo	Fever, amebas	Hepatotoxicity [stem]	[37]

Smilacaceae	*Smilax aristolochiifolia *Mill.	Zarzaparilla	Syphilis, psoriasis	Gastritis [whole plant]	[19]

Solanaceae	*Capsicum annuum *L.^*∗∗*^	Chile	Analgesic	Dermatitis by rubbing or gastritis, hemorrhoids, and colitis by oral administration [seeds, fruits]	[35]
	*Cestrum fasciculatum* (Schltdl.) Miers	Hierba del perro	Vomiting	Nausea and vomiting, dizziness [aerial parts]	[10]
	*Cestrum nocturnum *L.	Dama de noche	Headache, stomachache	tachycardia, dyspnea, fever, hallucinations [leaves]	[4]
	*Datura inoxia *Miller.^*∗∗*^	Toloache	Diabetes, asthma, wound healing	Narcotic, anorexic, cardiotoxicity, blurred vision [seeds]	[15]
	*Solanum americanum* Mill^*∗∗*^	Quelite mora	Headache, wound healing	Dermatitis, vomiting, severe diarrhea, paralysis [fruits]	[29]
	*Solanum elaeagnifolium* Cav.	Trompillo	Rattlesnake bite	Dermatitis [whole plant]	[13]
	*Solanum mammosum *L.	Chichigua	Diuretic, cough	Narcotic, cardiotoxicity [fruits]	[35]
	*Solanum nigrescens* M. Martens & Galeotti^*∗∗*^	Hierba mora	Fever, rheumatism	Immobilization [leaves and seeds]	[7]
	*Solanum rostratum* Dunal	Duraznillo	Diuretic, stomachache, diarrhea	Dermatitis [aerial parts]	[13]
	*Witheringia solanacea* L'Hér.	Merengena	To purify blood	Nausea and vomiting [leaves and seeds]	[47]

Urticaceae	*Urera baccifera* (L.) Gaudich. ex Wedd.^*∗∗*^	Ortiga brava	Diuretic, rheumatism, body pain	Dermatitis, edema [aerial parts]	[11]
	*Urtica mexicana* Liebm.	Ortiga	Diabetes, rheumatism	Skin burns [aerial parts]	[10]

Verbenaceae	*Duranta repens *L.	Mohuite de huerto	To purify blood, fever	Nausea and vomiting, seizures [flowers]	[4]
	*Lantana camara* L.^*∗∗*^	Hierba de San Pedro	Stomachache, diarrhea, rheumatism	Hepatotoxicity, nausea, and vomiting [aerial parts]	[52]
	*Lippia dulcis* Trevir.	Hierbabuena dulce	Cough, diarrhea, stomachache	Vertigo, hypotension [aerial parts]	[18]

Zygophyllaceae	*Larrea divaricata* Cav.^*∗∗*^	Chaparral	Rheumatism, tuberculosis, snake bite	Hepatotoxicity [aerial parts]	[56]
	*Larrea tridentata* (DC.) Coville^*∗∗*^	Gobernadora	Diabetes, diuretic	Hepatotoxicity, nausea and vomiting, abortifacient, gastritis [aerial parts]	[37]

*∗∗* indicates plant with toxicological information obtained from preclinical or clinical studies.

**Table 2:** Evaluation of causality and exclusion of alternate causes in clinical cases of medicinal plants from Mexico and Central America considered as toxic.

Information of the patient (age, gender, country of residence)	Plant, way of administration, dose and part of the plant consumed, time of consumption if indicated	First symptoms (onset, in days, of the first symptoms)	Toxic effects (onset, in days, of the toxic effects)	Clinical complications	Evaluation of causality (score)	Amount of information	Outcome (days)	Alternate causes excluded	Reference
9, M, Israel	*Jatropha multifida* Oral: >10 fruits	Vomiting, watery diarrhea, and abdominal pain (1 h)	Gastroenteritis (1)	Hepatic enzymes elevation	Naranjo (5): probable	Enough	Recovered (5)	ND	[43]
8, M, Israel		Vomiting, watery diarrhea, and abdominal pain (1 h)	Gastroenteritis (1)	Hepatic enzymes elevation	Naranjo (5): probable	Enough	Recovered (5)	ND	

20 children; 8–13 years old, India	*Jatropha curcas, *Oral: 1–4 seeds	1-2 h	Vomiting (95%) Headache (40%) Fever (40%) Diarrhea (50%)		ND	Regular	Recovered (6 hours)	ND	[57]

4, M, United States of America	*Conium maculatum* (piperidina 850 Ug/g plant), ND	Drowsiness (0.5 h)	Central nervous system depression (3 hours)	ND	Naranjo (6): probable	Regular	Recovered (6)	ND	[58]

19, F, Turkey	*Conium maculatum, *ND	Headache, blurred vision, and difficulty speaking (0.5 h)	Central nervous system depression (ND)	Difficulty breathing	Naranjo (5): probable	Regular	Recovered (1)	ND	[59]

5, M, India	*Argemone mexicana* Oral: oil	Abdominal pain, loos of movements and swelling throughout the body (20)	Epidemic dropsy (ND)	ND	Naranjo (6): Probable	Regular	Fatal (4)	Thalassemia and sickle cell disease (postmortem)	[60]
10, M, India		Abdominal pain, fever, and shortness of breath (20)		ND	Naranjo (6): Probable	Regular	Fatal (11)	Thalassemia and sickle cell disease (postmortem)	

2, F, Mexico	*Chenopodium ambrosioides, *Oral: oil 80 ml (1,560 mg ascaridol)	Vomiting and CNS depression (3 h)	Multiple organ dysfunction syndrome (ND)	Seizures, periods of apnea	Naranjo (6): probable	Enough	Fatal (3)	Encephalopathy due to lead poisoning and organophosphates	[61]

64, F, United States of America	*Cimicifuga racemosa, *Oral: capsules (80 mg/day/2 months)	Painful nodules on her left foot (ND)	Cutaneous vasculitis (42)	ND	Naranjo (5): Probable	Regular	Recovered (90)	ND	[62]
54, F, United States of America	*Cimicifuga racemosa* Oral: capsules (80 mg/day/4 months)	Ulcers (ND)	Cutaneous vasculitis (90)	ND	Naranjo (4): Possible	Regular	Recovered (90)	ND	

57, F, United States of America	*Cimicifuga racemosa, *Oral: capsules (without more specifications)	Lethargy and fatigue (14)	Autoimmune hepatitis (21)	ND	RUCAM (6) probable (Hepatocellular) Naranjo (7): Probable	Enough	Recovered (14)	Hepatic disease, serology of negative hepatitis, normal Antinuclear antibodies	[63]

16, F, United States of America	*Podophyllum peltatum* Topical; 20% of the resin in tincture	Vomiting, watery diarrhea, and abdominal pain (7 h)	Multiple organ dysfunction syndrome (<1)	Neurological toxicity and respiratory complications	Naranjo (6): probable	Enough	Recovered (120)	ND	[64]

18, F, United Kingdom	*Podophyllum peltatum, *Topical; 25% of the resin in tincture (7.5 ml = 1.88 g)	ND	Hypokalemia and peripheral neuropathy (<1)	Hypokalemia and peripheral neuropathy	Naranjo (5): probable	Regular	Recovered (90)	Previous cesarean anesthesia not discarded	[65]

21, F, United States of America	*Caulophyllum thalictroides, *ND	ND	Nicotinic toxicity (<1)	Tachycardia, diaphoresis, abdominal pain, vomiting and muscle weakness	Naranjo (5): probable	Poor	Recovered (1)	ND	[66]

40, M	*Thevetia peruviana* Oral: seeds	ND	Atrioventricular block (1)	Hyperkalemia, bradycardia	Naranjo (3): Possible	Regular	Recovered (3)	No cardiac history or vascular risk factor	[67]
25, F	*Cinchona pubescens* Oral: leaves	ND	Atrioventricular block (1)	bradycardia	Naranjo (3): Possible	Regular	Recovered (2)	ND	
75, F	*Cinchona pubescens* Oral: infusion	ND	Multiple organ dysfunction syndrome (ND)	ND	Naranjo (3): Possible	Poor	ND	ND	

20 patients: 9–50 years old, Mexico	*Metopium brownie, *ND	1–4	Erythema (95%) Vesicles (60%) Papules (4%) Blebs (2%)		ND	Regular	Recovered (ND)	Poison ivy	[68]

27, M, United States of America	*Larrea tridentata, *Oral: 500 capsules (3–7/day/10 months)	Vomiting, watery diarrhea, and abdominal pain (ND)	Hepatic damage (ND)	ND	Naranjo (4): Possible RUCAM (4): Possible (Hepatocellular)	Regular	ND	Hepatic disease, serology of negative hepatitis, (cytomegalovirus positive, ingestion of other plants and history of alcohol abuse)	[69]

13, M, Siri Lanka	*Abrus precatorius* Oral: 1 seed	Vomiting, watery diarrhea, and abdominal pain (5)	Pulmonary edema associated with hypertension (1)	Early renal parenchymal disease	Naranjo (5): probable	Enough	Recovered (3)	Organophosphate poisoning, acute glomerular nephritis, viral myocarditis, and dengue	[70]

17, F, India	*Abrus precatorius, *Oral: 10 seeds	Gastrointestinal (4)	Idiopathic intracranial hypertension (6)	Hepatic failure, hyponatremia, and hypokalemia	Naranjo (5): Probable	Enough	Recovered (21)	ND, intentional intake	[71]
28, F, India	*Abrus precatorius, *Oral: 7 seeds	Gastrointestinal (20)	Idiopathic intracranial hypertension (4)	Seizures and respiratory failure	Naranjo (5): Probable	Enough	Fatal (4)		

18, M, United States of America	*Datura inoxia*, Oral: seeds	Incoherences and hallucination (ND)	Anticholinergic intoxication (<1)	ND	Naranjo (4): Possible	Enough	Recovered (4)	Methamphetamine intake, and intake of other unknown substances	[72]

Age is given in years old; ND, not described; F, female; M, male.
